# Tinnitus groups: A model of social support and social connectedness from peer interaction

**DOI:** 10.1111/bjhp.12386

**Published:** 2019-08-26

**Authors:** Helen Pryce, Tiago Moutela, Colette Bunker, Rachel Shaw

**Affiliations:** ^1^ Department of Audiology School of Life and Health Sciences Aston University Birmingham UK; ^2^ British Tinnitus Association Unit 5 Acorn Business Park Sheffield UK; ^3^ Department of Psychology School of Life and Health Sciences Aston University Birmingham UK

**Keywords:** social connectedness, social support, tinnitus, tinnitus support groups

## Abstract

Tinnitus is a chronic condition for which there is no medical treatment. Tinnitus groups are a widely available resource for people with tinnitus.

**Objectives:**

Our objectives were to explore the active ingredients of tinnitus support groups in terms of their mechanisms for providing support, the contextual factors that elicit such mechanisms, and the outcomes in terms of coping enhancement.

**Design:**

We adopted a pluralist and iterative approach informed by the realist evaluation method.

**Methods:**

We conducted ethnographic data generation at tinnitus support groups involving observations (*n *=* *160), focus groups (*n* = 130), and individual interviews (*n *=* *20). Inductive analyses were conducted following the constant comparison method of grounded theory. We then interrogated the inductive themes to identify evidence of Contexts, Mechanisms, and Outcomes. We then produced a model which was tested in a survey of tinnitus group members (*n *=* *65) in effect providing large‐scale respondent validation of the data‐driven model created through our inductive analysis.

**Results:**

We identified that tinnitus groups can facilitate social connectedness between group members. This experience appeared to build resilience among those experiencing tinnitus‐related distress. Groups also played a role in building a sense of control related to knowledge and information sharing. Additionally, we identified risks associated with not accessing social support in a group environment.

**Conclusions:**

Our findings contribute to the growing understanding of the power of social connectedness as building shared social identity when living with tinnitus.

**Statement of contribution**

***What is already known on this subject?***

- Tinnitus is a prevalent condition with approximately 10–15% of the population experiencing a spontaneous sound without obvious source.
- Tinnitus is an invisible health and chronic condition. People with tinnitus experience high levels of distress, anxiety, and depression.
- Group support is beneficial to people with many health problems.

***What does this study add?***

- This study describes the mechanisms by which tinnitus support groups can support coping in tinnitus.
- This is the first study to comprehensively explore the views of those who attend tinnitus groups.
- The study identifies the key features of support groups that facilitate social connectedness among group members.
- The most valued features of groups are the knowledge and information provided, the sense of belonging communicated to group members, and the creation and maintenance of a sense of hope towards the tinnitus.
- This study contributes new insights to both the tinnitus field and adds to the literature on support groups in health.

## Background

Tinnitus is the experience of a spontaneous sound or ringing in the ears without obvious source (McCormack, Edmondson‐Jones, Somerset, & Hall, 2016) affecting approximately 10–15% of the population. Tinnitus is associated with reduction in quality of life (Dobie, 2003; Tyler & Baker, 1983) and with increased anxiety and depression (Dobie, 2003; Henry & Wilson, 1996).

Current interventions to help patients manage tinnitus focus on improving coping through therapeutic activity (Henry, Zaugg, Myers, Kendall, & Turbin, 2009). These activities are usually carried out in clinical settings, and in the United Kingdom, they are funded by the National Health Service (Hoare, Gander, Collins, Smith, & Hall, 2012). Support groups are an established way of helping people to cope with chronic health conditions. These may be led by peers or professionals, organized by charities, health service providers, or lay groups. Charitable support organizations provide a range of interventions for tinnitus, including support groups (Henry *et al*., 2009). These groups provide opportunities for information‐giving (Henry *et al*., 2009), peer support (Dibb & Yardley, 2006), and developing individual coping strategies (Dibb & Yardley, 2006).

In a systematic review of clinician‐led tinnitus management, audiologist‐led groups were identified to produce a moderate reduction in tinnitus severity (effect size 0.54; 95% CI 0.12–0.96; Hoare, Kowalkowski, Kang, & Hall, 2011) and contact with clinical staff produced greater changes in tinnitus severity scores than self‐help approaches (Hoare *et al*., 2011). However, the quality of this evidence is compromised by the lack of a consistent outcome measure. This reflects the complexity of existing tinnitus interventions and underlines the need to understand better the mechanisms that contribute to patient benefit Greenwell, Sereda, Coulson, El Refaie, & Hoare, 2016).

In other areas of health care, chronic disease is better managed by structured peer support and self‐management than by self‐management alone (Peterson & Clark, 1986). Group support is beneficial to people with many health problems (Bailer *et al*., 2004; Litt, Kadden, Cooney, & Kabela, 2003; Morley, Eccleston, & Williams, 1999; Simpson, Carlson, & Trew, 2001; Turner, 1982). Benefits are thought to arise from the opportunity to clarify appraisals of the health threat by making comparisons with other people who share the same condition. Making social comparisons improves coping potential (and so reduces distress); furthermore, the opportunity to receive social support from peers directly improves coping (Dibb & Yardley, 2006). Previous qualitative explorations of tinnitus groups employed interview methods to explore social support mechanisms. This work indicated that social support and the process of upward and downward comparison may be useful in re‐evaluating personal experience of tinnitus (Thompson, Pryce, & Refaie, 2011).

A particular challenge to the status of a person with tinnitus is the invisible and medically unexplained nature of the condition. This challenges medicalized scripts from clinicians which can lead to well‐intentioned reassurances that nothing is medically wrong and that patients just need to ‘learn to live with it’. These comments can be perceived as unhelpful to patients as they appear to invalidate the distress experienced (Pryce & Wainwright, 2008; Pryce *et al*., 2018). In this context, tinnitus groups can offer valuable support to self‐esteem. In order for the presence of others to provide support and reduce stress responses, a social identity ‘fit’ is required of the group members (Haslam, Rothschild, & Ernst, 2000). Research has not yet shown how the tinnitus support groups work to support individuals with their experience of living with invisible and medically unexplained persistent symptoms.

Tinnitus support groups are a complex intervention with a number of key features. Firstly, group members select membership of the group over other forms of coping behaviour. This means that the population who engage with groups are fundamentally different from those who do not, and this influences the likely impact of the group. Self‐categorization theories have proposed that in order for a group to have a coherent identity, there has to be a shift in individual self‐perception from a personal identity to a social identity (Turner, Oakes, Haslam, & McGarty, 1994). To understand the social function of tinnitus support groups, we needed an approach that focused on identifying the mechanisms that contribute to the groups’ function and which prioritized the subjective experience of its members. To achieve this, we used a realist approach. Our starting point was to inductively develop an understanding of the mechanisms by which the groups operated, to examine the contextual factors that facilitated these mechanisms, and to explore the range of outcomes in terms of coping responses that individuals had. We conceptualized tinnitus groups as social systems in which there is an active interplay between individual, institutional, micro‐, and macro‐social processes (Pawson, Tilley, & Tilley, 1997). In this study, we examined the individual‐level responses to the tinnitus group (the outcome), the mechanisms by which these outcomes were created (what happened during the group), and what contextual features were important in determining the way the mechanism operated (what contexts were required). Our aim was to extend beyond theory development or description to explanation by creating a data‐driven model of tinnitus support groups.

Our research question was:

- What features of tinnitus support groups facilitate coping?

Specifically, we aimed to:

- Identify mechanisms through which groups provide support for individuals living with tinnitus.
- Define the contextual factors upon which the mechanisms were dependent.
- Describe the outcomes that were derived from tinnitus groups.
- Identify features of coping that the groups facilitate.

## Methodology

### Design

**Figure 1:**
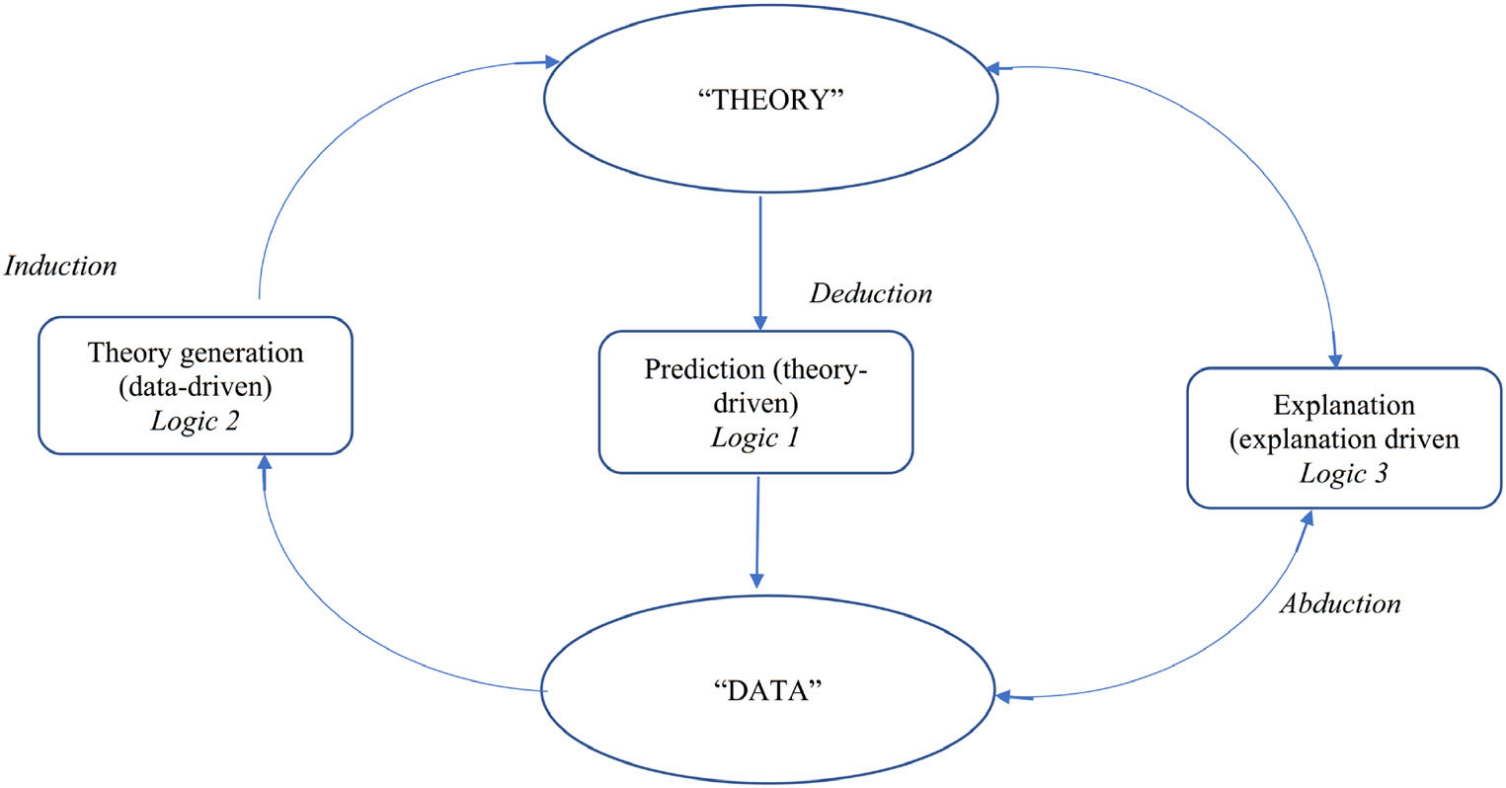
Logics of inquiry involving inductive, deductive, and abductive reasoning (adapted from Shaw *et al*., 2018)

We took a pluralist (Frost & Nolas, 2011) and iterative approach so we could respond to the particularities of each support group we encountered and so we could engage in a staggered, cyclical process of recruitment, data collection, and analytic activity in order to generate a theory grounded in the data (Strauss & Corbin, 1998). We drew on Shaw, Hiles, West, Holland, and Gwyther (2018) logics of inquiry (see Figure 1). We began in an exploratory mode, assuming a data‐driven logic of inquiry committed to theory generation. This is a typical vantage point from which to start exploratory qualitative work. The research team included a representative from the British Tinnitus Association (BTA) who could identify tinnitus groups and variations that existed in their delivery (to guide recruitment). The research was led by a researcher and hearing therapist with expertise in tinnitus and grounded theory research, with experience spanning over 20 years; a health psychologist with over 20 years’ experience of qualitative research; and a postgraduate research assistant with advanced training in health psychology and qualitative research methodology.

**Figure 2:**
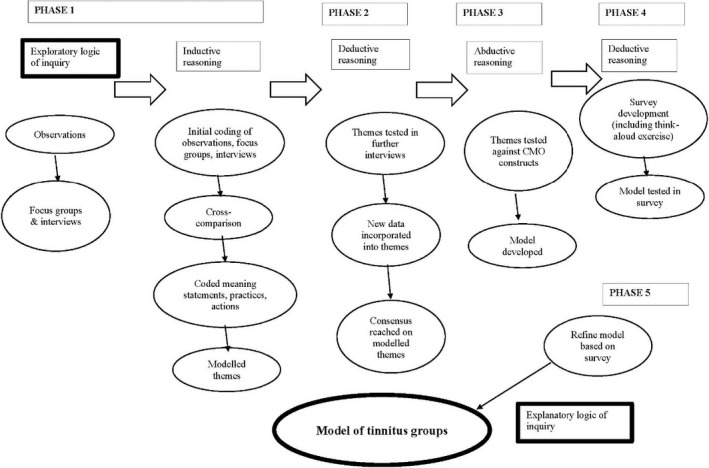
Flow diagram of methodological approach

We then moved through a series of steps, outlined below, involving a number of data collection methods and analytic reasoning approaches (see Figure 2). The results of this pluralist and iterative methodology were expressed in a model of tinnitus groups, thus finishing with an explanatory logic of inquiry.

### Participants

We approached tinnitus groups across the United Kingdom including all constituent countries, rural, semi‐rural, suburban, and urban settings. The BTA hold a record of all tinnitus groups that they are aware of, and we actively sought contrast in the group organizers, constituent membership, and activity from this list. The BTA support the groups through training and organizer support. Several groups do not engage with these offers, and we visited both those who engage with the BTA offerings and those that did not. We approached group leaders to participate, and where they volunteered, a visit to include consent taking was agreed. In keeping with grounded theory approaches, we purposefully sampled group locations to provide demographic contrast, contrast in group size, facilitation, and timing (to ensure we had access to group members who worked during the day). During visits, information sheets were provided to all members and researchers (HP &TM) visited the group to take consent. The study was approved by Aston University Life and Health Sciences Ethics Committee (ref: 1136).

### Phase 1: Exploratory data gathering and inductive reasoning

Our first stage of data collection borrowed from ethnography and involved observations during support groups, followed by focus groups with attendees of the groups. We also conducted individual interviews if that was more appropriate. The semi‐structured schedule (Table 1) acted as a guide for focus groups and interviews. Individual interviews and focus groups were audio‐recorded, and field notes provided a record of observations.

**Table 1:** Interview schedule

Interview schedule
Tell me about this group
How did you first get involved in the group? What makes you keep coming?
How does this group help you?
Is there anything that you would like the group to do differently? Does the group make your tinnitus worse in any way?
What would you say to someone who was thinking about joining a tinnitus group?

Field notes captured room layout, group dynamics, how participants moved through the space, who spoke when, and how conversation between other group members was managed by the group organizer (Emerson, Fretz, & Shaw, 1995). The researchers (HP &TM) recorded observations every 60 s to ensure all activities and actions within the group were captured.

Data gathered were analysed using inductive reasoning, allowing the data to lead initial coding. Following the steps of the constant comparison technique from grounded theory (Strauss & Corbin, 1998), we then compared codes across groups. The next stage of analysis involved chunking the coded data into coded meaning statements, practices, and actions. These coded data were then modelled into themes.

### Phase 2: Deductive reasoning and additional data gathering

Following the grounded theory approach, themes generated in phase 1 were checked in interview data generated in phase 2 and new data were incorporated into the existing themes. Adjustments were made to the themes to ensure they appropriately represented the full set of ethnographic data taken from observations, focus groups, and interviews. A consensus was reached within the team (HP, TM, RS & CB) on the themes to be taken forward into phase 3.

### Phase 3: Abductive reasoning using the constructs of Context, Mechanism, and Outcome

Abductive reasoning is an activity which involves a dialogue between the data and theory (Shaw *et al*., 2018). We took each theme and interrogated it for evidence of Contexts, Mechanisms, and Outcomes (CMO). This helped us organize the inductively generated themes pragmatically in order to answer the research question. From this, we developed a model of the CMO constructs which explained the findings we had generated.

### Phase 4: Checking the model against survey data using deductive reasoning

A survey was developed using the model created in phase 3. In consultation with tinnitus group members, researchers (HP & TM) identified quotations that best summarized elements of the model. We proposed these to the wider research group (HP, TM, CB, RS) and discussed as a group which quotation would be likely to be the clearest to rate on a scale of agreement. The quotations that were agreed upon formed version 1 of the survey. We then took the survey to a tinnitus group to check readability and usability using the ‘think‐aloud’ technique. Researcher TM asked group members to ‘think aloud’ as they completed the items. This technique revealed that items were intelligible and that the underlying concepts were recognized and understood. Following this piloting, we refined the survey by simplifying one quotation and removing one item. The items were finalized following one further consultation with additional support group members. The survey was then distributed to tinnitus support group members from groups we had visited and groups we had not visited. The findings from the survey data were analysed using descriptive statistics to provide an insight into a wider level of agreement with the key components of our model. As such, the survey functioned as a validity check by providing large‐scale member validation.

### Phase 5: Production of an explanatory model of tinnitus groups

**Figure 3:**
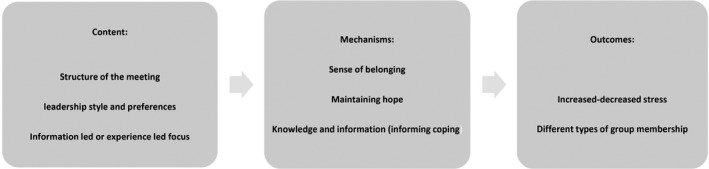
The process by which groups operate

The final phase involved refining the model based on the survey results and checking its explanatory power across all the data gathered and analyses conducted. We then produced the final version of our data‐driven explanatory model of tinnitus support groups (see Figure 3).

## Results

The groups visited enabled observations of 160 participants, focus group data from 130 participants, and individual interviews with 20 group organizers. A summary table describing the variations in the groups visited is presented in Table 2.

**Table 2:** Participating tinnitus groups and their characteristics

Group location	Location number	Type of organizers	Approx size of membership	Type of data gathered
City location in rural county of England	1	Lay	15–20	Observations and interviews with organizers
Small town South Wales	2	Professional – social care	4–6	Interview leader and focus group members
Semi‐rural South West England	3	Professional – NHS hearing therapist	6–8	Observations and focus group
City South West England	4	Professional – NHS audiologists	15–25	Observations, focus group, and interview organizers
London borough	5	Professional – private sector audiologist	6–10	Observations, focus group, and individual interview leader
Major city England	6	Lay	5–15	Observations and focus group
Major city England	7	Lay	5–15	Observations, focus group, and individual interview leader
Small town Midlands, England	8	Professional – private sector audiologist	15–25	Observations, focus group, and individual interview leader
Small town Midlands England	9	Lay	5–10	Observations and focus group
Affluent east Midlands town	10	Professional – private sector audiologist	40–60	Observations and focus group
City eastern England	11	Professional – NHS audiologist	10–20	Observations and focus group
Town South East England	12	Professional – NHS audiologist	8–15	Individual interview leader
Northern Ireland city	13	Professional – social support workers	12–15	Observations, focus group, and individual interview leader
Northern Ireland city	14	Professional – social support workers (sensory support team)	10–15	Observations and focus group
Northern town England	15	Lay	14–20	Model was tested using the survey
Scottish city	16	Lay	15–18	Observations, focus group, and individual interview leader
Midlands small town England	17	Lay	10–15	Model was tested using the survey

### Findings from interviews, focus groups, and observations

#### Contextual features

Groups that we observed varied in their membership, content emphasis, and organization. Groups had common features in that there was an organizer who provided some form of structure or focus to the meetings. Membership numbers ranged from 4 to 50. The groups sat together in a public space (church buildings, event rooms in pubs, and hotels were most common) and engaged in discussions about current tinnitus experiences, personal histories of tinnitus experiences, and involved general talks on the subject of tinnitus.

#### Context: Different types of members

We identified different types of membership to the group which were enacted by frequency of attendance and qualitatively different attachments to the group. We identified two categories of membership, the ‘in and out’ members and the ‘core group’ members.

When first joining a group, people have expectations of what they expect from the group meeting. These relate to the structure of the meeting, the members, and the dynamic of the group itself. Members suggested to us that if these expectations were not met, people would not return. Returning for future meetings was related to what is shared, and the way it is shared, compared against members’ expectations.

There were also descriptions from members about the impact of distress expressed by other members on their likelihood of returning to the group. Too much distress was seen as incompatible with group benefit.

There was a woman at the first or second meeting I went to at [location] who was in a very similar situation. I mean she was in a terrible state. I don't think she got out of that meeting what she really wanted. (male participant, location 11)

Some people who attended support groups (especially people who were attending for the first time) seemed to go to the meeting hoping to find a cure, and to stop going as soon as they realized that might not be possible.

I think what they want is instant relief. They think if they come here then we'll wave a magic wand and the tinnitus will be gone. I actually was asked if I could cure the tinnitus and I say, ‘No, I'm very, very sorry, I wish I could,’ but the only thing we can do is we can give you loads of information and we can support you. We can listen to you, the group will listen to you. (group facilitator, location 9)

The contextual features varied within the range outlined in Table 3.

**Table 3:** Variation and spread of key group features

Key contextual features	Variation range from	Variation to
Membership	In and out membership	Core group attachment
Organizers	Directive and leading	Facilitating and inclusive
Content	Information led	Experience led

#### Context: Variations in emphasis within groups

##### Information‐giving groups

The key aspect of these groups is that they give priority to speakers and focus on information about tinnitus. Rather than focusing on having members sharing their experiences, these groups focus on having a guest speaker talking about a subject area, and then having the members discussing what they heard and how that was relevant to them.

Members associate the positive aspects of these groups with the quality of the information: *The knowledge, I came here for the knowledge (male participant, location 11)*. The type of guest and the way they approached the group and delivered the talk were also particularly relevant. *But sometimes you get clinical researchers who are made for the lab, and the office. They're not public speakers… too much medical jargon (male participant, location 8)*.

Regardless of their positives, groups that only focus on the informational role and lacked opportunities to share experiences had limitations:

That's what I found useful. I mean the [speaker] couldn't give us any answers, but information he shared. But people's experiences, I think we lack that a little bit. (male participant, location 10)

The groups operated in each case with a clear sense of ‘ingroup’ and ‘outgroup’. In group characteristics included a consistent experience of tinnitus, a degree of distress from tinnitus, and the impact of tinnitus on everyday life.

I think we can do that (share bad experiences with the group) because we know that everybody understands. When I talk to people outside, they're not interested because they can't relate to it. (female participant, location 8)

However, it is important to note that there might be risks associated with group membership.

I've got a friend on the forum and she only went to her local group (…) she only went once because everybody was crying, so she never went again. (female participant, location 8)

##### Experience‐sharing groups

The key aspect of these groups was that they prioritized members sharing their experiences and tended to focus on the emotional side of things. These groups tended to be less formal, and their focus went beyond tinnitus: *Don't just focus on tinnitus because that's sort of grinding it in isn't it? (male participant, location 9*).

we all sit down, we all usually ask each other how we've gone through the week and then once we've had a little discussion or a chat, then we start going into other things (…) anything in the papers or politics. (male participant, location 8)

Even family members of people with tinnitus attending these groups recognized the positive aspects of meetings where members discussed diverse topics that did not always focus on the experience of living with tinnitus.

What I think, as an outsider living with tinnitus, how this group operates by sometimes you diversify. You may only talk about tinnitus for ten minutes during the one hour and I think that does good because it brings everybody out of themselves and they're talking about other things. (husband of a group member with tinnitus, location 8)

##### Information‐giving and experience‐sharing groups

There were some groups which offered a combination of both information‐giving and experience‐sharing. These meetings were normally divided into two parts: firstly a guest speaker and secondly a chance to share experiences and exchange ideas. Sometimes groups ran one session entirely based around experience‐sharing followed by a meeting purely focused around a talk given by guest speaker. Sometimes this was due to group leader preference or difficulties identifying relevant guest speakers that would make a positive contribution to the group meeting.

I see it as coming to see a speaker and then, talking to people afterwards. So, that's how I see it. And I think that's what it needs to be. Because you can talk to anybody about it. (male participant, location 11)

So, I do sort of pay attention to everything that's happening [talks given by guest speakers]. And also listening to other people. And personally, I didn't hear anybody really moan today about it. You know? I tried to give a backstory, for an understanding. And I think it's useful if other people do the same. (male participant, location 11)

#### Context: Variations in group organizer

Regardless of their professional background, group organizers were key to determining the emphasis of the group. Within our observed groups, approximately one‐third of the group organizers were professionals within audiology and two‐thirds were individuals directly affected by tinnitus (either as a person with tinnitus or as a close family member). Those that had a professional role included audiologists, hearing aid dispensers, and hearing therapists. Group organizers assumed responsibility for the practical running of the group, the way the group communicated, and the content of sessions. In every case, group organizers were volunteering their time in addition to other professional or voluntary roles. In addition, they worked to provide information to group members, invited speakers, and dealt with distressed group members.

Whatever I can find on the internet, I print it off (…) that's another thing, as much information as you can get. (group facilitator, location 9)

This infers a degree of expertise on behalf of the organizers who engaged with information and the speakers they invited. We observed variation of additional support provided by group organizers.

[group organisers] they're controlling it in a way in who they invite. And what the message is coming across, and then they know what the content is backwards and forwards. (male participant, location 11)

Some group leaders saw their role solely as organizers, whereas others went as far as providing peer support counselling, visiting people in their homes, or providing support over the phone to members in distress.

I think it's on everybody that they know when they do come in and they say something, it stays here and if people ask for help, we have one‐to‐one and we get through that. We have one‐to‐one and we help as much as we can. (group facilitator, location 9)

Yes, I have had to stay on the phone with people suffering from tinnitus for hours, just chatting and talking about life and tinnitus (group facilitator, location 16)

### Mechanisms: How the group provides support

The tinnitus groups helped define the experience of tinnitus as discrete and constructed the person experiencing it as part of a particular social group. Membership of the group is an important mechanism for developing a social identity as someone with tinnitus.

Group members displayed empathic communication to each other and encouraged discussion about an otherwise private and little discussed experience. This is particularly valuable when living with a medically unexplained and invisible condition. People living with medically unexplained health conditions have particular difficulty in identifying as someone with a diagnosis. Without a visible health condition, the legitimacy of human suffering is not socially condoned or justified (Ferrari & Kwan, 2001). Membership of a tinnitus group affords this social status. Rather than playing the passive ‘sick role’ (Parsons, 1975) of the patient, recipient of care, sufferer, or (in some cases) victim, the individual proactively joins a shared social identity as people coping with tinnitus.

This provides a sense of meaning, purpose, and belonging to the individual and is evidenced by examples of people wanting to retain membership of the group to support others.

I'm ok – it doesn't affect me anymore. It's a great privilege to be able to sit here. It's one of things tinnitus has given me to be able to meet people with tinnitus. It's a massive positive because tinnitus has given me like that. (male participant, location 3)

### Mechanisms: Informing coping

The tinnitus group provided a coherent depiction of coping with tinnitus. This was evidenced in the descriptions given by group members of their daily lives with tinnitus. It was negotiated in the upward and downward comparisons that formed part of the group discussions about their experiences of and feelings towards tinnitus.

lots of people got it but not as bad as me (male participant, location 7)

He's at the stage I was at two years ago. So I've just gone up and had a word with him and told him what I went through and suggested what he might go through, which he was very grateful for. And we haven't had a lot of that, of people talking about their experiences… (male participant, location 9)

### Mechanisms: Constructing a social identity – ‘The same as I am’

Individual stories provided an opportunity for social comparison which was described as helpful.

I find that I can relate to it more because I feel the same at times, the way that they do. I know how odd and difficult at times you get and listening to people feel the same, it just makes me feel like somebody else out there who has got it, who is suffering the same as I am. (male participant, location 8)

On the other hand, if negative stories dominated a meeting, this could have a negative impact on some members who were struggling to cope with their tinnitus. This seemed to have a particular impact on people who were attending a meeting for the first time.

Well, one of the reasons I avoided it, as a matter of fact, going to a group was because my sister‐in‐law had tinnitus. And went to group and everyone was saying how bad it was and how terrible they felt (male participant, location 9)

The feature that differentiated these reactions appeared to be the presence or absence of resonance and identification with an individual as ‘someone like me’. However, if an individual (frequently during our observations a new comer to the group) was exposed to the same sort of story and did not identify with that person, it would be experienced negatively.

hearing this, this is the worst night of my life. (male participant, location 5)

In this way, for those who experienced a sense of belonging, the group provided social capital to the identity of someone with tinnitus. Some groups invited external agencies to provide information or, in some cases, to sell a treatment. The presence of these external agencies inferred status on its members as ill persons that were worthy of their time and offerings. Most importantly to the group members, this conferred a sense of having knowledge. Regardless of whether the information communicated was accurate or relevant to the individual, it conferred a sense of being informed which appeared more important than the information itself. When questioned, group members rarely described a new insight into tinnitus that was obtained from the tinnitus group, for example, *I'm finding out the latest things (female participant, location 5)*. Rather, they described a valuable sense of being informed, and being on the frontline for any eventual cure information.

### Mechanisms: Maintaining hope

Having access to knowledge seemed to build resilience to the tinnitus.

It's given me an educational perspective (female participant, location 5)

Within the group, the shared resilience to negative ideas or experiences was a valuable re‐enforcer of status and worth.

We need to know things at our level, what we can do, what's support is there out there. What kind of cognitive behaviour treatment, all this type of thing. Then that gives you a little bit of hope there is something out there. (male participant, location 9)

Hope was a key feature of tinnitus support groups. Hope was enacted by ‘success stories’, that is, by group members describing an improved sense of coping with their tinnitus.

The other thing that helped me as well, I used to like reading success stories, knowing that there was, that it wasn't always going to be like this, hopefully. It gave me hope and it's like when I came in, I saw people who were living with it and getting on with it. Like Joyce, she's had it for all those years, but she's still enjoying life. People like that. (female participant, location 8)

So, if you get a bunch of positives, yeah. It's going to help, isn't it? (male participant, location 9)

### Mechanisms: Sense of belonging

A sense of belonging was communicated repeatedly in both interviews and verbal and non‐verbal communications observed within the groups. Belonging to a group and the feeling of ‘being surrounded by those who understand’ underlined the importance of being part of a group.

I find coming and talking about it and listening to other people has helped me a lot. Not thinking you're just on your own, you're the only one who has got it. (male participant, location 8)

Takes the pressure off knowing that there's someone like yourself there. (male participant, location 9)

The sense of belonging is further empowered through the sharing of ‘wonder treatments’. There was little scrutiny of treatment claims and little discussion of science, which left the groups vulnerable to misinformation. This was regulated by group consensus, and group organizers neither condoned nor refuted claims of treatment successes of new drugs or devices. The thrust of this narrative observed in groups was to ‘give it a go’.

there are cures for it. I've known two people take these herbs that they were given and they both worked. (female participant, location 5)

This is inherently dangerous and risks harm. Promoting misinformation with expensive or potentially harmful ‘treatments’ without criticism can render group members more vulnerable. It also risks the credibility of information shared within groups, thereby discrediting those offering an information‐giving service.

The themes presented represent the complexity identified in the construction of tinnitus support groups. Figure 3 summarizes the contextual and mechanistic themes and illustrates how they relate to the outcomes experienced. This model indicates how each theme operated to determine successful group membership.

#### Survey results

Following the inductive thematic modelling of tinnitus support groups, we identified three key mechanisms:

- Knowledge and information
- Sense of belonging
- Hope.

We invited a further 11 groups to complete the survey, which yielded 65 responses from nine groups. Results indicated a wide level of agreement with these key mechanisms. Table 4 details the breakdown of responses.

**Table 4:** Survey responses

Survey item	Theme	Agree or strongly agree	Neither agree nor disagree	Disagree or strongly disagree
Talking about tinnitus and listening to other people has helped me a lot	Sense of belonging	62	3	0
The group will listen to you	Sense of belonging	61	4	0
I think it is important just to be in a room with other people who suffer the same condition	Sense of belonging	62	2	0
I came here for the knowledge	Knowledge and information	62	3	1
If people know there is a speaker they are more likely to attend	Knowledge and information and membership variation	54	8	2
A few people pass through the group	Membership variation	38	22	2
GPs know about tinnitus groups	Knowledge and information	9	18	36
You need structure to the meetings	Knowledge and information	45	9	8
If it was a specialist coming in, just giving facts then I wouldn't come	Knowledge and information	27	12	26
If you are a new member then you don't want to hear the negative side of things all the time	Hope	34	13	14
Other people's stories give me hope	Hope	54	9	0

The survey generated agreement with the themes generated through inductive analysis. The survey responses offered a high level of agreement with the themes of knowledge and information, sense of belonging, and hope. There was a reasonable agreement that membership varied between individuals and that while speakers were valued, there needed to be opportunity for peer support as well.

## Discussion

This evaluation of the context, mechanisms, and outcomes of tinnitus support groups has identified that groups assist individual members to develop and refine a social identity as someone with tinnitus. For those who become members, tinnitus support groups facilitated their coping mechanisms by providing social support. The ‘core group’ membership experienced by some reinforced this social identity through a sense of belonging. Furthermore, being a tinnitus group member seemed to adjust the ontological features of tinnitus itself. In other words, group membership inherently altered members’ experiences of their own tinnitus and what it meant for them to be someone with tinnitus. The contextual features of the groups – whether information‐giving, experience‐sharing, or both – functioned to either facilitate or obstruct this creation of social support.

One key variation in group outcome was the variation experienced by ‘in and out’ group members. These members did not experience a sense of belonging and did not always experience the group positively. They provided ‘negative cases’ in which group membership did not provide social support. These individuals either engaged with the group solely as a means to obtain information or, in some cases, found that listening to others share experiences of difficulty with tinnitus enhanced their own distress with their tinnitus.

This highlights the important distinction between social support and social connectedness (Jetten, Haslam, Haslam, Dingle, & Jones, 2014). Social connectedness has been identified as the characteristic that reaches beyond friendly support towards a process whereby the identity of the individual shifts from an individual ‘I’ to a collective ‘we’ (Jetten *et al*., 2014). Being part of a collective group identity can foster resilience, which is important, especially for those living with invisible health conditions which are frequently described as socially isolating (Schaaf, Flohre, Hesse, & Gieler, 2014). Evidence from studies of people with depression highlights that a lack of social connectedness is associated with preceding depressive symptoms (Cruwys *et al*., 2013). The mechanisms behind this refer to Cooley's ‘looking glass self’ principles of self‐esteem and self‐identity (Cooley, 1902). People with invisible hearing conditions, such as tinnitus, benefit from social connectedness which can validate the tinnitus experience as part of their identity. For people with tinnitus, it seems that social connectedness can be an effective ‘social cure’ for the distress associated with tinnitus (Jetten *et al*., 2015). A key mechanism observed in tinnitus support groups is their ability to provide a forum for social connectedness and in turn enhancing coping strategies. Our findings suggest, in keeping with self‐categorization theory, that tinnitus support groups can provide a buffer to distress for those who choose to pursue membership.

### Limitations and implications

This is the largest study of its kind, and it has demonstrated a new theoretical understanding of tinnitus support groups. This extends existing research showing that upward and downward comparisons function as an effective mechanism for managing tinnitus well. It also suggests that the formation of a coherent social identity as a person with tinnitus is made possible by group membership. This social identity in itself builds resilience.

Our model was developed using grounded theory principles of constant comparison and checked using a survey as respondent validation. As such, the survey was designed to check existing theory and confirm emergent themes, and it was not intended as a stand‐alone investigation. Similarly, it is worth noting that attendees at a tinnitus group were able to provide some insights into reasons for attendance and non‐attendance, but further research with those who do not attend groups would enhance this. This study relied on a combination of research methodologies. Our overarching philosophy was derived from the realist approach and the assumption that there are multiple components that interact and contribute to an outcome. This approach has explanation at its core; for example, what works in tinnitus support groups, for whom, and under what circumstances? We acknowledge some tensions between the positivism of the realist approach and a grounded theory approach which straddles both inductive and deductive processes. Within the context of this study, taking this pluralist and iterative approach has enabled us to frame the social intervention of tinnitus support groups as a set of mechanisms and outcomes within the context of the social world.

Future tinnitus groups should take care to build social support, offer members a sense of belonging, and preserve hope in the everyday management of tinnitus. Together, these key features of this social intervention will provide a buffer to the stress of living with tinnitus. The role of a shared social identity in managing unseen health conditions is an important contributor to reducing suffering.

## Conflict of interest

None.
